# GJA8 missense mutation disrupts hemichannels and induces cell apoptosis in human lens epithelial cells

**DOI:** 10.1038/s41598-019-55549-1

**Published:** 2019-12-16

**Authors:** Li Li, Da-Bei Fan, Ya-Ting Zhao, Yun Li, Zi-Bing Yang, Guang-Ying Zheng

**Affiliations:** 1 Ophthalmologic Center, the First Affiliated Hospital of Zhengzhou University, Zhengzhou, 450052 China; 2 Endocrine Department, the First Affiliated Hospital of Zhengzhou University, Zhengzhou, 450052 China

**Keywords:** Next-generation sequencing, Hereditary eye disease, Genetics research

## Abstract

Autosomal dominant congenital cataract (ADCC), the most common hereditary disease, is a major cause of eye disease in children. Due to its high genetic and clinical heterogeneity, the identification of ADCC-associated gene mutations is essential for the development of molecular therapies. In this study, we examined a four-generation Chinese pedigree with ADCC and identified putative mutations in ADCC candidate genes via next-generation sequencing (NGS) followed by Sanger sequencing. A novel missense mutation in *GJA8* (c.T217C) in ADCC patients causes a serine-to-proline substitution at residue 73 of connexin 50 (Cx50); no mutation was found in unaffected family members and unrelated healthy individuals. Functional analysis revealed that this missense mutation disrupts protein function in human lens epithelial cells (HLEpiCs), which fails to form calcium-sensitive hemichannels. Furthermore, mutant Cx50 leads to decreased ROS scavenging by inhibiting *G6PD* expression and thus induces cell apoptosis via aberrant activation of the unfolded protein response (UPR). In conclusion, we report a novel *GJA8* heterozygous mutation in a Chinese family with a vital role in ADCC, broadening the genetic spectrum of this disease.

## Introduction

Congenital cataract (CC) primarily triggers lens disorders leading to infant amblyopia or blindness during the early development of vision^1^. According to preliminary statistics, nearly 1/3 of infant blindness worldwide is caused by CC^2,3^. Many cases of CC are induced by metabolic disorders, intrauterine infection and hereditary factors, among which autosomal dominant (AD), autosomal recessive (AR) and X-linked mutations are a major cause of CC. ADCC exhibits a high degree of clinical and genetic heterogeneity, and over 22 genes have been identified to date as being associated with ADCC^4–6^. For instance, a missense mutation in the DNA-binding domain of heat-shock transcription factor 4 was found to lead to ADCC in the British population^7^. In addition, Yu *et al*. identified two missense mutations in Cx50, Cx50P59A (c.175 C > G) and Cx50R76H (c.227 G > A), causing ADCC in a Chinese pedigree and observed cosegregation among affected individuals^8^. Interestingly, mutation analyses of six potential candidate genes associated with congenital cataracts, *CRYGC*, *CRYGD*, *CRYGS*, *GJA8*, *GJA3* and *CRYAA*, revealed three mutations that generate premature stop codons, thereby truncating the protein, in CRYGD to be associated with congenital cataracts^9^. Most of these genes are prominently involved in lens development, lens intercellular communication and lens fiber organization.

Connexin gap junction channel proteins are necessary for the intercellular transport of small biomolecules in the lens^10,11^. Hence, connexin is crucial for maintaining lens homeostasis and transparency. Connexin 50 (Cx50), encoded by the *GJA8* gene, is primarily expressed in lens fibers; Cx50 has been linked to ADCC by studies showing that membrane transport proteins are pivotal for lens embryological development^12,13^. GJA8 mutations typically lead to inefficient gap junction channel formation, abnormal subcellular distribution, altered channel and/or hemichannel function and changes in electrophysiological characteristics^8,14,15^. Hence, mutations in *GJA8* are responsible for ADCC.

Oxidative stress-induced reactive oxygen species (ROS) production is known to contribute to the development of various cataracts^16–18^, and cataract-associated proteins encoded by mutant genes can trigger cell apoptosis as a result of the unfolded protein response (UPR)^19–21^. The UPR is activated during lens development due to the accumulation of unfolded proteins or oxidative damage and can induce cataract formation by inhibiting protein synthesis and fiber cell elongation, resulting in cell apoptosis. UPR activation has already been observed with cataract-related proteins, especially the Cx50 mutant^19,21–23^. Therefore, studying *GJA8* mutations is essential for the molecular diagnosis of ADCC.

In the present study, we clinically examined a four-generation Chinese family affected by ADCC with congenital perinuclear cataracts and identified a novel heterozygous missense mutation in *GJA8* via next-generation sequencing, which we verified by Sanger sequencing. The novel missense mutation (p.S73P), in which a serine is substituted by proline, was the primary cause of ADCC in this family. *In silico* analysis revealed alterations in the structure and characteristics of the protein. Furthermore, we performed cellular experiments to dissect the pathogenic mechanism of this missense mutation and found that loss of hemichannel function and UPR activation might disrupt lens homeostasis and ultimately cause cataract formation. These findings extend our understanding of the molecular mechanism of ADCC and expand the mutational spectrum of Cx50 in Chinese ADCC patients.

## Results

### Clinical evaluation

**Figure 1:**
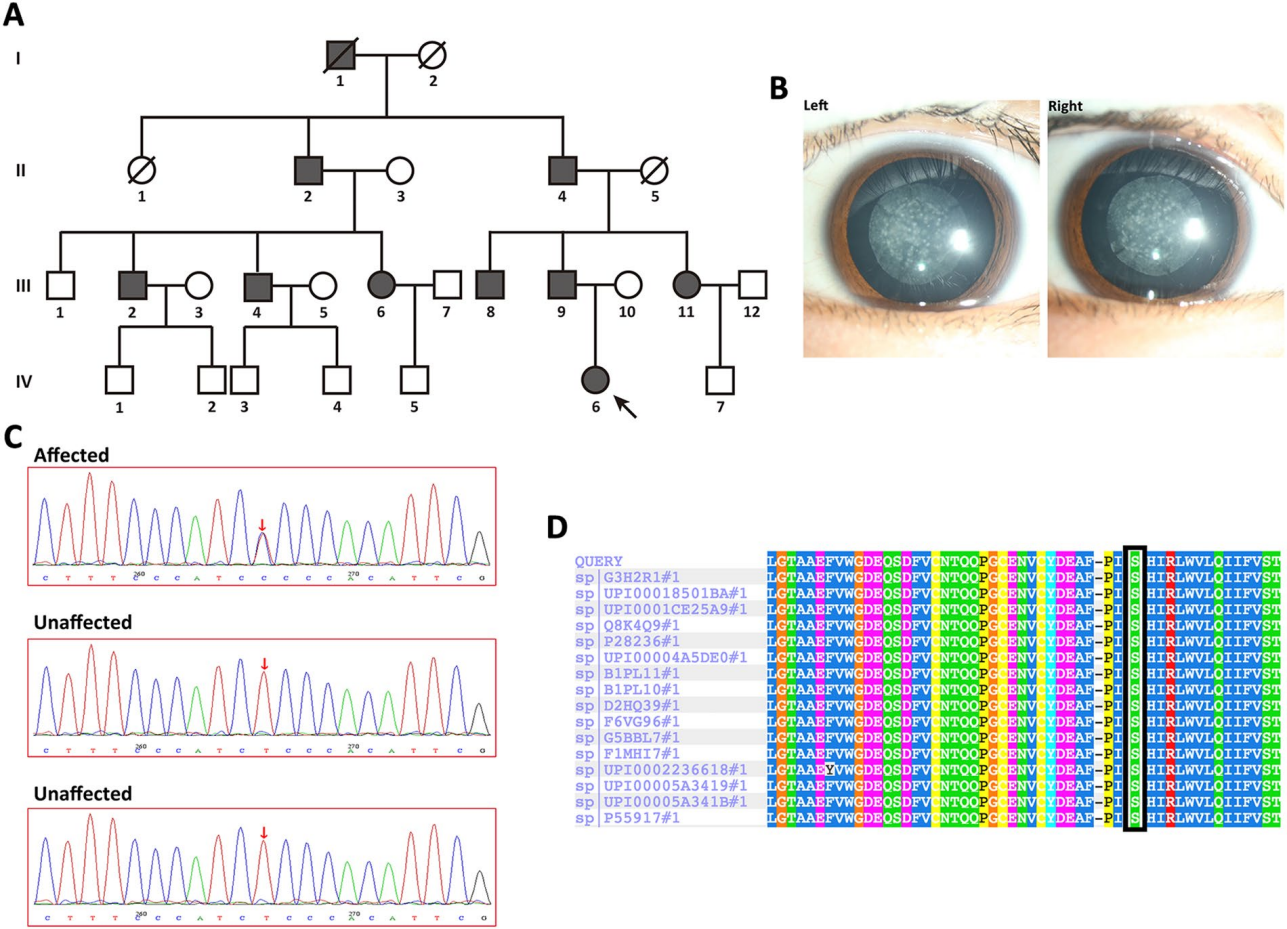
Cataract pedigree, clinical evaluation, mutation detection and multiple sequence alignment analysis of the *GJA8* mutation site. (**A**) Pedigree information of the four-generation family. ■ or ● represents cataract-affected members, and □ or ○ represents unaffected healthy members. ■ or □ represent males, and ● or ○ represent females. A black arrow indicates the proband. All living participants in this pedigree were involved in this project. (**B**) Slit-lamp photography of the proband’s eyes. (**C**) Mutation site screening of the *GJA8* gene by Sanger sequencing. The red arrow indicates the mutation site. (**D**) Multiple sequence alignment of Cx50 encoded by *GJA8* from various species. The black frame indicates the mutation site.

One Chinese family from Henan Province was recruited for this study. The pedigree of this family was plotted according to the clinical investigation, revealing an autosomal dominant inheritance pattern. This family spans four generations, with nine living affected members and thirteen living healthy members (Fig. 1A). After clinical evaluation, we found that the proband, a 5-year-old child, had congenital perinuclear cataracts (Fig. 1B) compared to a healthy eye (Fig. S1). Specifically, we observed opacities in the central nuclear lens of the eye. None of the other members of this family suffered from other related ophthalmic or systemic syndromes.

### One novel missense mutation was identified in ADCC patients

We sequenced 134 candidate genes using genomic DNA obtained from blood samples. We retrieved 570.85 Mb of raw data and 531.43 Mb of processed data from the NGS results. More than 95% mean coverage of target regions was obtained in this NGS dataset, with an average sequencing depth of >400×. The coverage of target bases for the N10 and N20 reads from this dataset was 85.3% and 75.6%, respectively. After filtering and validation, only one novel exon variant was found, in *GJA8* (RefSeq, NM_005267.5; Protein ID, NP_005258.2), and was identified in the proband as being associated with ADCC. This novel variant is a missense mutation at position 217 (c.217 T > C) that results in the substitution of the conserved serine with proline at codon 73 (p.S73P). In addition, we verified this mutation in all affected members by Sanger sequencing (Fig. 1C) associated with ADCC and found it to be absent in all unaffected family members and in healthy controls. Moreover, this variant was located in the GJA8 known to cause ADCC, demonstrating that this mutation is not an SNP or described previously by other groups. Therefore, this mutation is not a rare polymorphism in the population but rather a pathogenic mutation in this cataract pedigree.

### Bioinformatic assessment of mutation impairment

Four bioinformatic prediction programs were used to assess the structural and functional effects of the p.S73P mutation in Cx50. The three predictions by SIFT, PROVEAN and Mutation Taster revealed that this mutation is deleterious. Furthermore, we performed multiple sequence alignments to evaluate the conserved nature of this site and found that Cx50 serine 73 is a highly conserved site among several mammalian species (Fig. 1D). In addition, SWISS-MODEL prediction demonstrated that this mutation disrupts a hydrogen bond with residue 76 (Fig. S2A). Moreover, we examined the hydrophobicity of the wildtype and mutant proteins and found that p.S73P slightly alters the hydrophobicity of the protein (blue arrow, Fig. S2B). These bioinformatic data suggest that the missense mutation p.S73P is a deleterious mutation that leads to ADCC, warranting further experimental validation to unravel the effect of the identified novel mutation.

### Functional analysis using transfected cells

**Figure 2:**
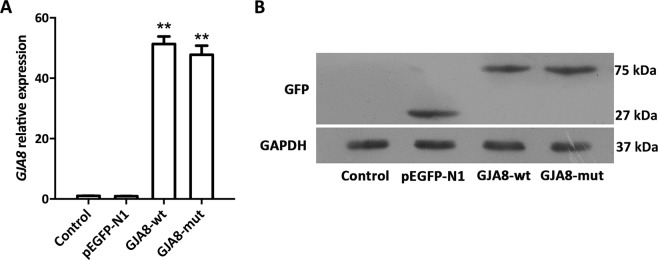
Molecular validation of wildtype and mutant Cx50 proteins in transfected HLEpiCs. (**A**) *GJA8* relative transcript abundance in control, pEGFP-N1-transfected, GJA8-wt-transfected and GJA8-mut-transfected cells. *GAPDH* was used as a housekeeping gene. (**B**) Western blotting of the Cx50 protein using an anti-EGFP antibody in control, pEGFP-N1-transfected, GJA8-wt-transfected and GJA8-mut-transfected cells. GAPDH was used as an internal control. The original image is provided in Supporting Information (Fig. S4).

We performed cell assays to verify the bioinformatics data. First, we transfected recombinant plasmids into HLEpiCs using Lipofectamine 2000 and seeded the transfected cells onto plates containing DMEM supplemented with G418. Relative *GJA8* expression levels were evaluated by quantitative polymerase chain reaction (qPCR). As shown in Fig. 2A, significantly higher *GJA8* expression was detected in transfected cells compared with nontransfected and EGFP-transfected cells. Protein expression was further determined by Western blotting using an anti-EGFP antibody detecting a band at 27, 75 and 75 kDa in empty vector- and wildtype and mutant GJA8-transfected cells, respectively, whereas no such bands were detected in nontransfected cells (Fig. 2B). These data suggest that genes with or without the mutation were successfully expressed in HLEpiCs.

### EGFP fluorescence observation of Cx50 distribution and aggregation

**Figure 3:**
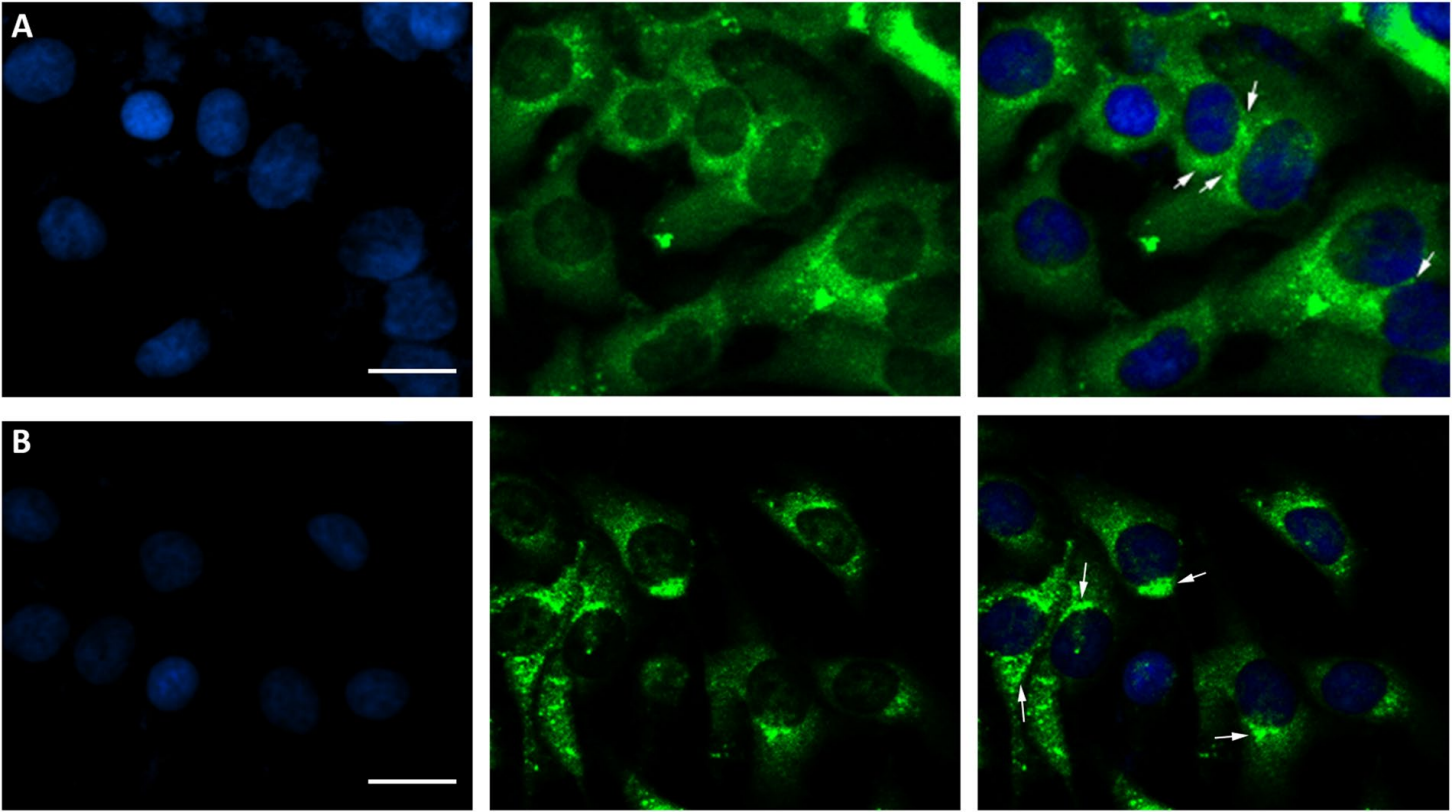
EGFP fluorescence of wildtype and mutant Cx50 proteins in HLEpiCs. From left to right, DAPI fluorescence, EGFP fluorescence, overlay image. (**A**) wildtype Cx50; (**B**) mutant Cx50. The white arrow represents the typical EGFP fluorescence. Scale bar, 10 μm.

Gap junctions consist of connexin protein subunits, and Cx50 has been shown to modulate the diffusion of molecules between lens fiber cells to regulate the electrical properties of the plasma membrane^24,25^. Hence, we first investigated the subcellular localization of both wildtype and mutant Cx50 in HLEpiCs to reveal the protein distribution and aggregation. Confocal microscopy revealed that wildtype Cx50 localized to the cytosol and the plasma membrane (white arrows), forming hemichannels; in contrast, the mutant protein aggregated in the cytosol, and no fluorescence was observed at the plasma membrane (Fig. 3). Our results demonstrate that the missense mutation p.S73P causes Cx50 to aggregate in the cytosol, thereby disrupting trafficking to the plasma membrane for gap junction formation.

### Effect of the missense mutation on cellular hemichannels

**Figure 4:**
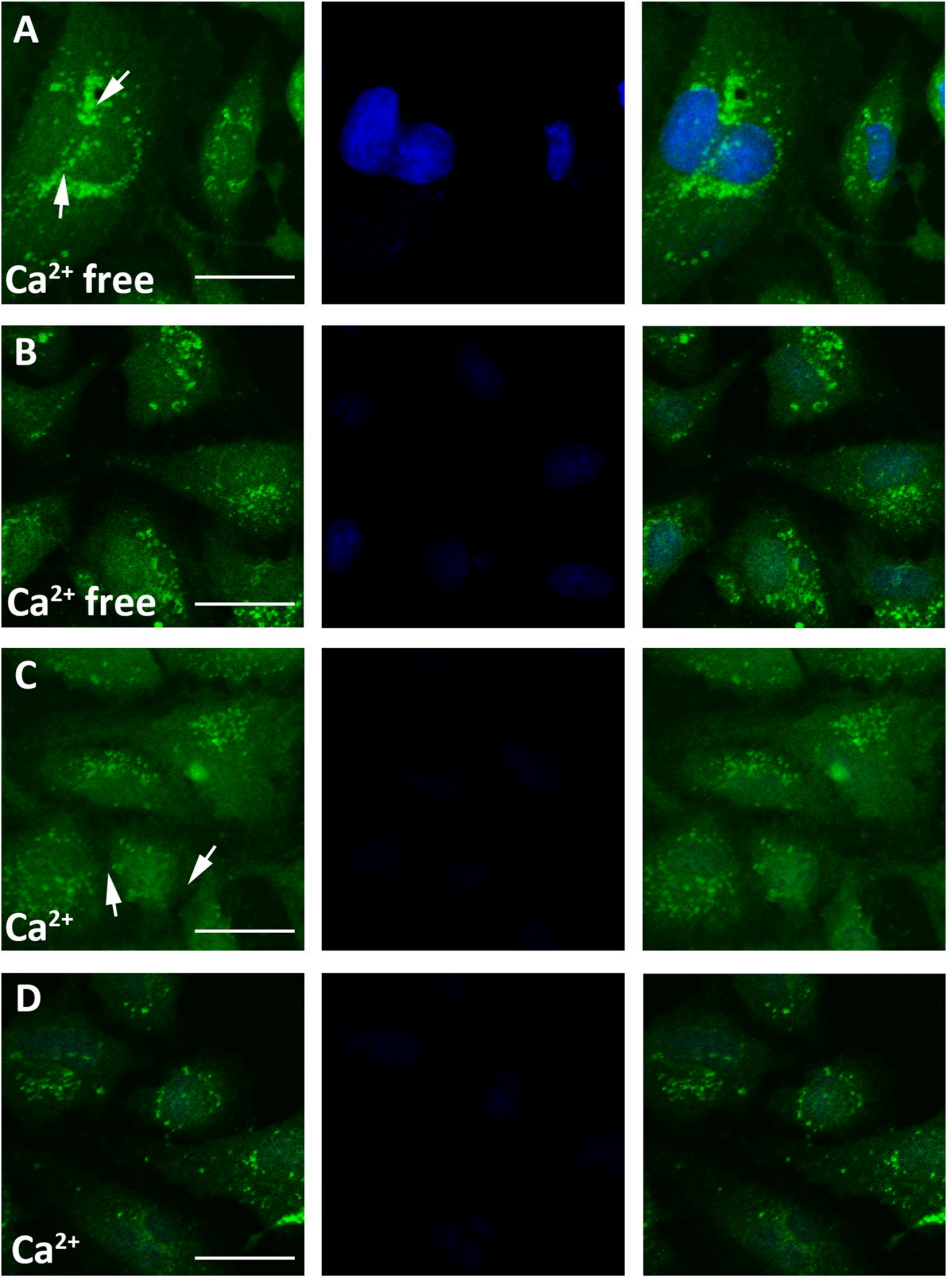
PI uptake by HLEpiCs. (**A**) Wildtype Cx50-transfected cells without Ca^2+^; (**B**) mutant Cx50-transfected cells without Ca^2+^; (**C**) wildtype Cx50-transfected cells with 1.2 mmol Ca^2+^; (**D**) mutant Cx50-transfected cells with 1.2 mmol Ca^2+^. From left to right, EGFP fluorescence, PI fluorescence, overlay. The white arrow represents the hemichannel region. Scale bar, 10 μm.

To detect the consequences of this mutation, we next examined hemichannel function. First, propidium iodide (PI) uptake experiments were performed under Ca^2+^-free conditions and in the presence of 1.2 mmol/L Ca^2+^ for the same period of time. As shown in Fig. 4, PI uptake confirmed that the most GJA8-wt-overexpressing cells contained high-intensity PI fluorescence localized in nuclei, though few GJA8-mut-overexpressing cells displayed blue nuclear fluorescence under Ca^2+^-free conditions. As expected, at 1.2 mmol/L Ca^2+^, both GJA8-wt- and GJA8-mut-overexpressing cells exhibited little nuclear PI staining, suggesting that the blockade of cellular hemichannels by high Ca^2+^ concentrations limited dye uptake.

Furthermore, we determined the membrane potential of HLEpiCs transfected with GJA8-wt and mut under Ca^2+^-free and 1.2 mmol/L Ca^2+^ conditions. As shown in Fig. S3A, the membrane potential of GJA8-mut-expressing HLEpiCs transfected with GJA8-wt was much lower than that of GJA-wt-expressing HLEpiCs (115 pA vs 325 pA). As expected, under 1.2 mmol/L Ca^2+^ conditions, the membrane potential of GJA8-wt HLEpiCs decreased and reached a similar value as that of GJA8-mut HLEpiCs. Our data demonstrate that the missense mutation causes loss of hemichannel function.

### p. S73P mutation-triggered cell death

**Figure 5:**
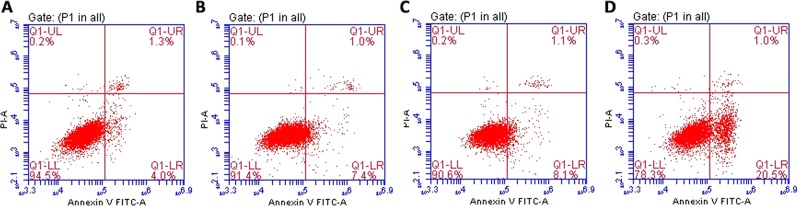
Apoptosis analysis by flow cytometry to detect (**A**) control cells, (**B**) cells expressing pEGFP-N1; (**C**) cells expressing the wildtype Cx50 protein, and (**D**) cells expressing the mutant Cx50 protein.

As mutations in connexin proteins have previously been reported to induce cell apoptosis^26–29^, we evaluated the viability of cells expressing this missense mutation. As shown in Fig. S3B, cell viability was significantly decreased after transfection of the plasmid expressing the mutant protein, demonstrating that cells overexpressing GJA8-mut might undergo cell death. Additionally, we determined the influence of the missense mutation on apoptosis via Annexin V-fluorescein isothiocyanate (FITC)/propidium iodide (PI) staining. Cells expressing with mutant Cx50 showed a significant apoptosis trend compared with wildtype Cx50-expressing and control cells (Fig. 5). These results indicate that the missense mutant causes cell apoptosis.

### Potential mechanism of cell apoptosis caused by GJA8 missense mutation

**Figure 6:**
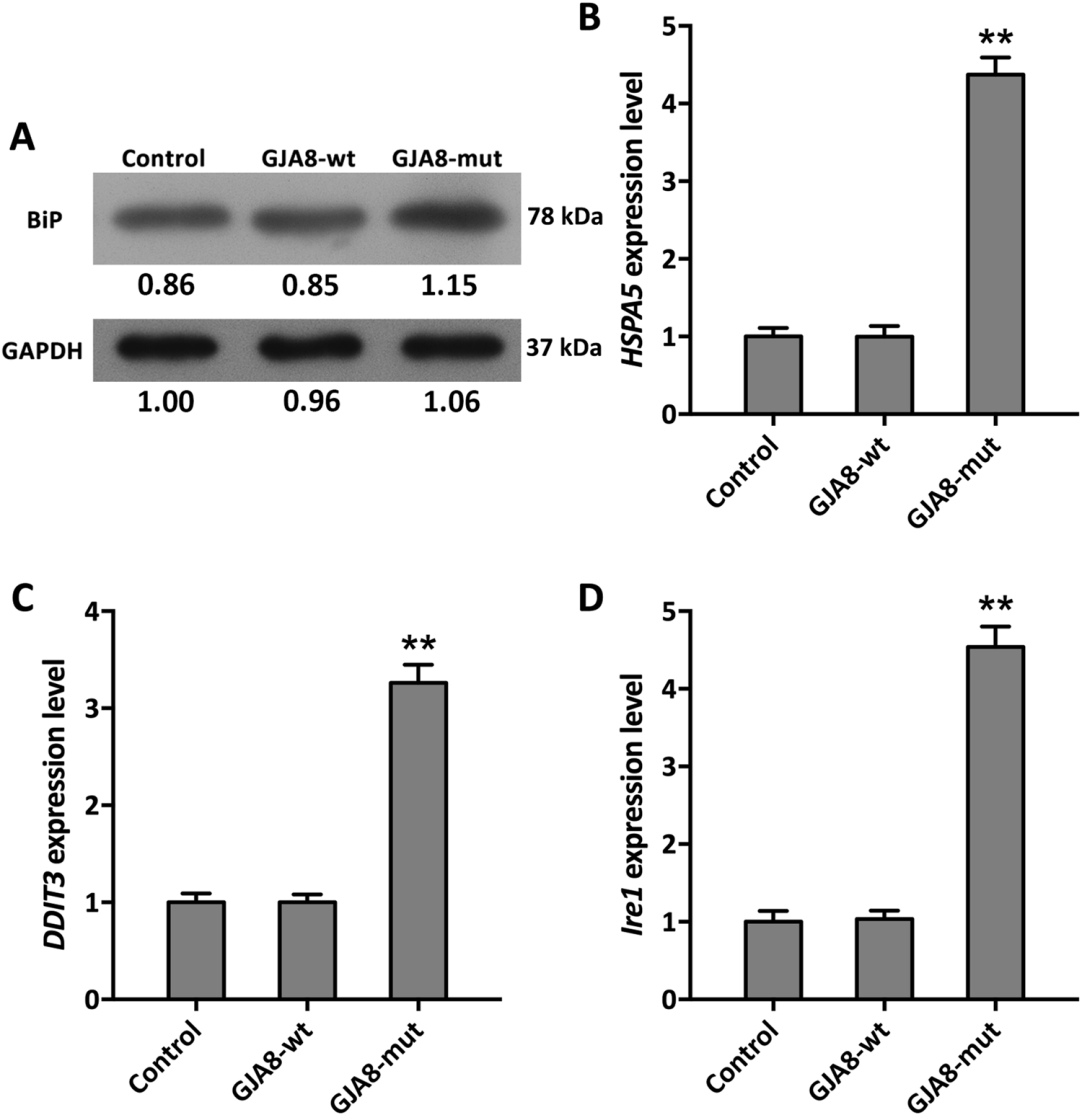
Expression levels of UPR-related genes. (**A**) BiP protein levels in transfected cells. (**B**) *HSPA5* transcript abundance in transfected cells. (**C**) *DDIT3* transcript abundance in transfected cells. **(D**) *Ire1* transcript abundance in transfected cells. The original image is provided in Supporting Information (Fig. S4).

The UPR plays a prominent role in the pathogenesis of protein aggregation in cataracts^23^, and Alapure noted that UPR activation occurred in Cx50-mutant mouse lenses^30^. Hence, we assessed the endoplasmic reticulum (ER)-targeted protein BiP, which strongly stimulates UPR activation. As expected, BiP expression was upregulated by mutant Cx50, as based on Western blotting (Fig. 6A). Furthermore, we detected the expression pattern of UPR-related genes, such as HSPA5 and DDIT3, and found that the relative transcript levels of both were significantly increased by 4.3- and 3.3-fold in cells expressing the GJA8-mutant compared to the control and GJA8-wt-expressing cells, respectively (Fig. 6B,C). However, there was no significant difference observed in terms of the gene expression level between the control and GJA8-wt-expressing cells. We previously reported that the transmembrane sensor Ire1 is activated via noncanonical splicing of X-box-binding protein 1 (Xbp1) mRNA; hence, we also measured Ire1 transcript levels^23^. As shown in Fig. 6D, the relative mRNA abundance of Ire1 was significantly elevated by 4.5-fold in the GJA8-mutant group compared to the control and GJA8-wt groups, whereas the relative transcript level of Ire1 similar between the control and GJA8-wt groups.

**Figure 7:**
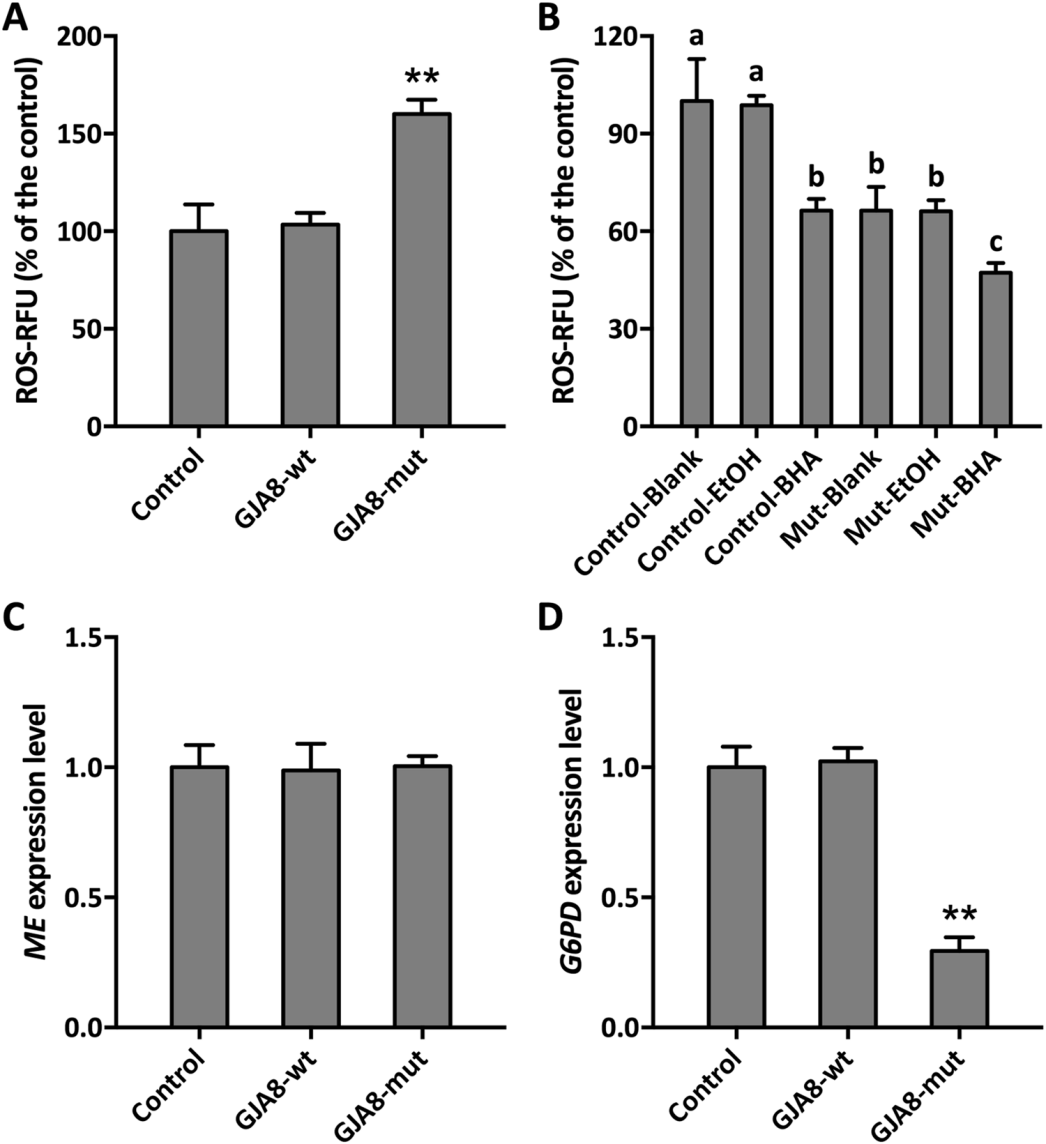
ROS levels and NADPH biosynthesis-related gene expression in transfected cells. (**A**) ROS fluorescence levels in transfected cells. (**B**) ROS fluorescence level in transfected cells treated with BHA or vehicle (EtOH). (**C**) *ME* transcript abundance in transfected cells. (**D**) *G6PD* transcript abundance in transfected cells. A significant difference is indicated at the *p* < 0.01(**) level. Different lowercase letters on the bars indicate significant differences at the *p* < 0.05 level. Each value represents the mean ± SD (*n* = 3).

We then determined cellular ROS levels to evaluate the effect of oxidative damage induced by the Cx50 mutant. Interestingly, ROS levels in the GJA8-mutant group were extremely high compared with controls, suggesting that ROS-induced BiP upregulation ultimately leads to apoptosis (Fig. 7A). To test our hypothesis, we further determined cell viability following treatment with the ROS scavenger butylated hydroxyanisole (BHA)^31,32^. As shown in Fig. S3C, cells of the GJA8-mut group exhibited increased survival in the presence of BHA compared with untreated cells, indicating that BHA-mediated ROS scavenging increased cell viability, which is consistent with the ROS levels observed upon BHA treatment (Fig. 7B). To determine how the Cx50 mutant triggers ROS production, two NADPH biosynthesis genes, namely, ME and G6PD, were examined by qPCR. Interestingly, ME expression levels were not affected in the GJA8-mut group (Fig. 7C), whereas G6PD levels were significantly decreased compared with the control and GJA8-wt groups (Fig. 7D). These data suggest that the Cx50 mutant induces G6PD inhibition via the pentose phosphate pathway (PPP), resulting in ROS generation and further leading to apoptosis through UPR activation.

## Discussion

CC is caused by multiple genes and represents a severe hereditary ophthalmic disease resulting in cataract disorders^33^. In recent years, high-throughput molecular techniques have substantially advanced our molecular understanding of the genetic basis of CC, and numerous mutations have been linked to CC^1,34,35^. Therefore, identification of gene mutations can be important for CC diagnosis. In the present report, we identified a novel missense mutation in *GJA8* in a four-generation Han family by NGS and Sanger sequencing. This mutation cosegregates in the patients in this pedigree, and no such mutation was found in healthy family members or in 100 healthy individuals. CC exhibits significant phenotypic and genotypic heterogeneity. Previous reports have reported this feature in various instances, showing that various gene mutations can result in uniform clinical features and that similar mutations may result in different phenotypes of different family members^7,36–40^ and revealing a lack of a considerable relationship between genotype and phenotype. Berry *et al*. demonstrated that a missense mutation (NM_001040667.2:c.190 A > G; p. K64E) in the DNA-binding domain of HSF 4 is associated with ADCC in the British population and observed cosegregation in all the affected family members^7^. Congruently, sequence analysis of the whole genome of individuals affected by cataract uncovered a splice-site mutation (c.2826–9 G > A) cosegregating with the disease^41^. Consistently, our pedigree analyses demonstrated that this novel *GJA8* missense mutation cosegregates with the disease. These results suggest that this mutation is responsible for the congenital perinuclear cataract phenotype in the Han Chinese population.

Ions and small solutes can easily pass through gap junctions; hence, intercellular gap junctions are vital for cellular processes^42,43^. Gap junctions consist mainly of three isoforms of connexin proteins, namely, Cx43 (encoded by *GJA1*), Cx46 (encoded by *GJA3*) and Cx50 (encoded by *GJA8*), in the vertebrate avascular lens to support lens homeostasis and transparency. Connexin proteins consist of an N-terminus, four transmembrane domains (M1, M2, M3, M4) linked a single cytoplasmic loop and two extracellular domains (E1, E2) and a C-terminus. Cx50 is a critical protein that plays numerous roles in lens growth, fiber maturation and transparency^44–46^. To date, nearly thirty novel mutations have been identified in Cx50 as being related to cataracts^47^, and most of these mutations are involved in ADCC and are located in the N-terminal half of Cx50. Interestingly, our mutation, which affects residue 73, is also near the N-terminus, within the first extracellular domain (E1) (Fig. S2C), implying the potential loss of GJA8 function caused by the loss of transmembrane structure. Tong *et al*. found that mutations in E1 are responsible for altering the unitary channel conductance of Cx50 gap junctions^48^. Moreover, multiple sequence alignments showed that position 73 in Cx50 is highly conserved across multiple species, suggesting the potential structural and functional importance of this site. In addition, the mutation was found to cosegregate with the affected ADCC patients, whereas no such mutation was present in unaffected family members or healthy controls. Similar to other mutations in Cx50^8,49,50^, this p.S73P mutation is predicted to be deleterious by several online bioinformatics tools, suggesting that might cause disease.

Connexins such as Cx50 normally form connexin hemichannels docked in adjacent cells, allowing transport across cell membranes^51^, and the phylogenetically conserved E1 domain regulates hemichannel integrity and is thus important for the transport between process^48,52^. The located of our mutation, p.S73P, in the E1 domain suggests a similar loss of hemichannel function. To verify this, functional analyses were performed to evaluate the consequences of this missense mutation. As shown in Fig. 2, qPCR analysis and Western blotting revealed similar mRNA and protein levels in wildtype and mutant GJA8-expressing cells, indicating no effect of the missense mutation on expression *in vivo*. Previous reports found that wildtype Cx50 localizes to the plasma membrane and appositional membranes, and EGFP fluorescence imaging showed wildtype Cx50 to be distributed in the cytoplasm and the plasma membrane in HLEpiCs and HeLa cells. In contrast, the mutant protein aggregated in the cytoplasm in HLEpiCs, and no obvious EGFP fluorescence was present at the plasma membrane between adjacent cells. This finding showed that the p.S73P mutation altered the subcellular distribution of Cx50, causing it to coaggregate in the cytosol, consistent with previously reported mutations located in the E1 domain being related to ADCC^48,53,54^. Indeed, a previous study confirmed that mutations in the Cx50 E1 loop result in loss of function with regard to functional gap junction channel formation^52^. Furthermore, we detected hemichannel function using dye-uptake assays, and the results confirmed our prediction that the aberrant protein leads to the loss of hemichannel function. This mutation might alter docking capability for gap junction channel formation, thereby blocking Cx50 trafficking to the plasma membrane and ultimately leading to lens metabolic disorder and cataract formation. Additional functional studies were then performed to clarify the mechanism of cataract formation caused by the p.S73P mutation. As connexin proteins regulate cell growth, which is related to cell proliferation^55^, we first detected cell proliferation and found that p.S73P mutation had a negative effect on cell growth. Additionally, flow cytometry analysis of Annexin V-FITC/PI staining indicated cell apoptosis caused by the missense mutation. Previous studies reported that abnormal hemichannel change caused by a mutation in *GJA8* resulted in cell death^27,29^. In the present study, p.S73P caused loss of hemichannel function, which appeared to be the main reason for cell apoptosis, suggesting that Cx50 p.S73P-induced loss of hemichannel function eventually triggers apoptosis.

Misfolded proteins can cause endoplasmic reticulum stress, which triggers the UPR, leading to apoptosis^56^. The UPR is an adaptive intracellular signaling response to aberrant protein production^57^. Normally, UPR activation in cells induces expression of a series of related genes, and HSPA5, DDIT3 and Ire1 are key players in UPR signaling^19,58,59^. Figure 6 shows that the UPR was activated by our misfolded protein, suggesting that UPR stress was the major pathway leading to cell apoptosis. The G98R mutation in αA-crystallin causes UPR stress, resulting in cell apoptosis^60,61^. Similarly, the Cx50 mutant triggered abnormal protein expression, leading to the transcriptional activation of UPR genes and inducing apoptosis and cataract development^21,25,27^. Moreover, ROS levels in cells overexpressing the mutant protein were higher than in control cells, demonstrating that ER stress caused ROS production^29^. Intriguingly, cells treated with the ROS scavenger BHA displayed downregulated ROS levels and increased cell viability. These results suggest that the mutation resulted in ROS overproduction and then induced UPR to cause apoptosis. NADPH, the main cofactor involved in ROS scavenging, is synthesized by the products of the *ME* and *G6PD* genes. We found that the Cx50 mutant led to downregulation of *G6PD*, which is involved in the PPP pathway, leading to decreased ROS scavenging ability and elevated ROS levels and leading to UPR activation and cataract formation.

In conclusion, the present study identified a novel missense mutation in *GJA8* that caused apoptosis and resulted in congenital perinuclear cataract development. Our data provide insight into the mechanism of the misfolded protein formation caused by the p.S73P mutation. Additionally, our findings extend the mutation spectrum of ADCC-causative genes in the Chinese population.

## Materials and Methods

### Ethics statement

This study was performed in accordance with the ethical guidelines of the Declaration of Helsinki and was approved by the Institutional Review Board of the First Affiliated Hospital at Zhengzhou University (Zhengzhou, China). Appropriate written informed consent was obtained from all participants or their pediatric legal guardians.

### Clinical examination

One Chinese family of Han ethnicity affected by ADCC from Henan Province was recruited from the Ophthalmology Department at First Affiliated Hospital of Zhengzhou University. At least 100 unrelated healthy participants without eye disease from Henan Province were chosen as healthy controls. The related medical history of the affected family was recorded. Venous blood samples were collected from the proband and family members and placed in tubes containing ethylenediaminetetraacetic acid (EDTA).

### Next-generation sequencing and sanger sequencing

Genomic DNA from the proband was extracted for next-generation sequencing from blood samples using QIAamp DNA Blood Mini Kit (Qiagen, USA) according to the manufacturer’s instructions. All candidate genes associated with CC and other ophthalmic diseases based on the Online Mendelian Inheritance in Man (https://www.ncbi.nlm.nih.gov/omim) database were investigated in this study, following a previous study^23^; details of the related genes are provided in Supplementary Dataset 1. Library preparation was performed following standard protocols described previously. Target exon sequence capture was accomplished using biotinylated oligonucleotide probes and a disease-associated gene panel according to the manufacturer’s instructions. Paired-end sequencing for 100-bp reads was performed using the Illumina HiSeq. 2000 platform (Illumina, USA).

Raw sequence reads were first filtered by Trim-Galore to export clean data aligned to the human genome by the BWA program. The quality of the clean reads was recalibrated and realigned to reference using GATK software. In addition, duplicated reads were removed by Sequence Alignment/Map tools 3 (SAMtools 3). Unique mapping reads were then applied for variant detection annotated by in-house bioinformatics tool with RefSeq (hg19, from UCSC) and UCSC annotation according to the manufacturer’s recommendation. After bioinformatics analysis, the SNPs/indel were filtered with a frequency of above 5% by databases, including the 1000 Genome database (http://www.1000genomes.org/), Exome Variant Server database (https://evs.gs.washington.edu/EVS/), Human Gene Mutation database (http://www.hgmd.cf.ac.uk/ac/index.php) and dbSNP database (http://www.ncbi.nlm.nih.gov/SNP/). One probable causative *GJA8* variant (Gene ID 2703, OMIM 600897, candidate gene of congenital cataract) was identified by NGS analysis and further verified by Sanger sequencing. DNA was extracted from blood samples from the family members, including affected (n = 9) and unaffected (n = 13) individuals, and healthy unrelated controls (n = 100). PCR was performed using predesigned primers, and the products were purified using a fragment purification kit. The PCR products were inserted into pUC19 for sequence analysis using an ABI DNA Analyzer (ABI, USA). The effects of the mutation were predicted using online bioinformatics tools such as PROVEAN (http://provean.jcvi.org/seq_submit.php), Mutation Tasting (http://www.mutationtaster.org/) and SIFT (http://blocks.fhcrc.org/sift/SIFT.html). The hydrophobicity of the wildtype and mutant proteins was predicted using Protscale. In addition, the 3D structure of the wildtype and mutant proteins was analyzed using an online tool (SWISS-MODEL, https://swissmodel.expasy.org/).

### *GJA8* plasmid construction and cell transfection

Control human lens epithelia cells (HLEpiCs) from an unaffected subject were obtained from American Type Culture Collection (ATCC) and cultivated in Dulbecco’s modified Eagle’s medium (DMEM, Gibco, USA) supplemented with 10% fetal bovine serum (FBS, Gibco, USA). The cells were cultured at 37 °C in a humidified 5% CO_2_ atmosphere.

Recombinant human *GJA8* was amplified from genomic DNA and inserted into the pEGFP-N1 vector. The mutant *GJA8* gene was obtained using a Site-Directed Mutagenesis Kit (Vazyme, China). Recombinant plasmids containing the normal or mutant gene were transfected into HLEpiCs using Lipofectamine 2000 (Invitrogen, USA) according to the manufacturer’s protocols.

### Molecular validations of transfected cells

To verify transfection, we performed qPCR and Western blotting as described previously^62^. The amplification efficiency of the primers was determined before performing qPCR, and only primers with greater than 95% efficiency were used for further analysis. The housekeeping gene *GAPDH* was employed to normalize the threshold cycle (Ct) of the transfected and control cells. Western blotting was performed to detect Flag-tagged protein expression levels. Cells were lysed in RIPA buffer for protein extraction. Proteins were detected using a primary anti-EGFP antibody (1:2000, Abcam, USA) and normalized to the internal control GAPDH with anti-GAPDH antibody (1:3000, Sangon, China).

### Fluorescence microscopy of Cx50

Cells transfected with recombinant plasmids were used for fluorescence microscopy. Briefly, transfected cells were washed three times with pre-chilled phosphate-buffered saline (PBS) for 10 min at 4 °C and incubated with DAPI for 10 min at room temperature for nuclear staining. Afterwards, the cells were observed using a fluorescence microscope to examine the Cx50 protein distribution. The protein distribution and aggregation in cells were calculated from 200 positively transfected cells in at least 10 randomly observation fields.

### Dye uptake analysis

For hemichannel activity analysis, transfected cells were washed with Ca^2+^-free HBSS and incubated with 0.1% propidium iodide (PI) for 30 min. The cells were washed three times with PBS buffer and observed using a confocal laser microscope as described previously^13^.

### Cell proliferation assay

The cell counting kit 8 (CCK-8, Dojindo, Japan) assay was performed to determine the viability of both wildtype and mutant Cx50-expressing cells. Specifically, cells transfected with the plasmid expressing wildtype or mutant Cx50 were cultured for 48 h in RPMI 1640 medium containing 10% fetal bovine serum and then seeded into 96-well plates at a density of 5 × 10^3^ cells per well. Thereafter, 10 μL of fresh CCK-8 solution was added, and the plate was incubated for 4 h at 37 °C. The optical density (OD) at 450 nm was measured for each well using a microplate reader.

### Cell apoptosis analysis

Cell apoptosis was evaluated using an Annexin V-FITC/PI Cell Apoptosis Detection Kit according to the manufacturer’s recommendations with a BD FACS Aria IIu apparatus and Cell Quest software (BD biosciences, USA); H_2_O_2_ stimulation for 24 h was performed.

### Molecular validation of UPR-induced apoptosis in Cx50 mutant cells

qPCR was performed to quantify expression of UPR-associated genes following the protocol provided above. The ribosomal protein gene *Rp119* served as a housekeeping control. Western blotting was also performed as described above using an anti-BiP/Grp78 (1:2500, Abcam, USA) primary antibody.

### ROS determination in Cx50-mutant cells

We determined ROS levels using the cell-permeable fluorogenic probe 2′7′-dichlorfluorescein diacetate DCFH-DA (Sigma, USA) according to previous report^63^. Briefly, cells in 24-well plates were washed twice and then incubated with 10 mM DCFH-DA for 30 min at 37 °C. Fluorescence intensity was detected using a microplate reader (Bio-Tek, USA) at 485 nm excitation and 520 nm emission.

To examine NADPH biosynthesis in Cx50-mutant cells, the expression levels of two dominant genes, *ME* and *G6PD*, involved in NADPH biosynthesis were measured as described above.

### Statistical analysis

All experiments in this study were carried out in at least three biological replicates. Statistical analyses were performed using the SPSS statistical package. Student’s t-test was performed to compare wildtype with the different groups. Difference with were considered significantly different at *p* < 0.05 (*) or *p* < 0.01 (**). The data obtained were subjected to Kruskal-Wallis one-way Analysis of Variance (ANOVA) followed by Duncan’s method. Different lowercase letters on the bars indicate significant differences at *p* < 0.05 level.

## Supplementary information

Supplementary Information

Supplementary Information
