# Supplementary material for: GJA8 missense mutation disrupts hemichannels and induces cell apoptosis in human lens epithelial cells
Source: Sci Rep. 2019 Dec 16;9:19157. doi: 10.1038/s41598-019-55549-1 (PMC6915756; doi:10.1038/s41598-019-55549-1)
Supplement: Supplementary file 1 — Supplementary Information [file 41598_2019_55549_MOESM1_ESM.docx]

**GJA8 missense mutation disrupts hemichannels and induces cell apoptosis in human lens epithelial cells**

Li Li^+1^, Da-Bei Fan^+2^, Ya-Ting Zhao^1^, Yun Li^1^, Zi-Bing Yang^1^, Guang-Ying Zheng*^1^

^1^Ophthalmologic Center, the First Affiliated Hospital of Zhengzhou University Zhengzhou 450052, China; ^2^Endocrine Department, the First Affiliated Hospital of Zhengzhou University, Zhengzhou 450052, China

Correspondence to G. Zheng: email: zzzgy01@126.com.

+These authors contributed equally to this work.

**
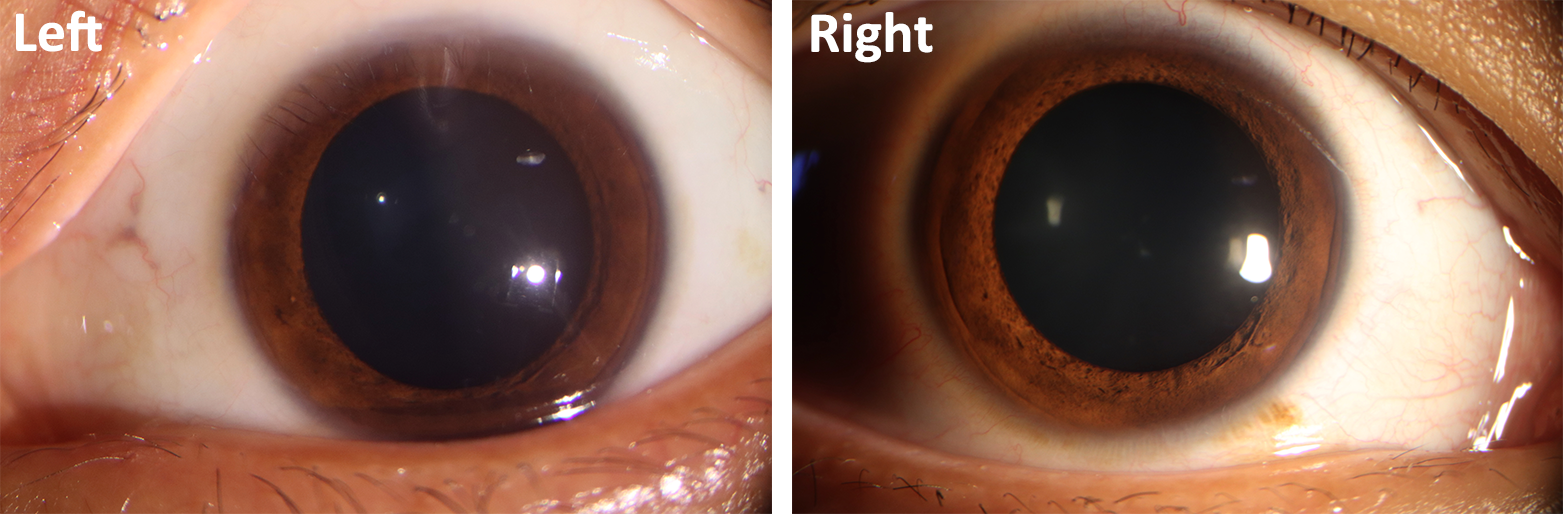
**

**Figure S1.** Slit-lamp photography of the healthy eyes.

**
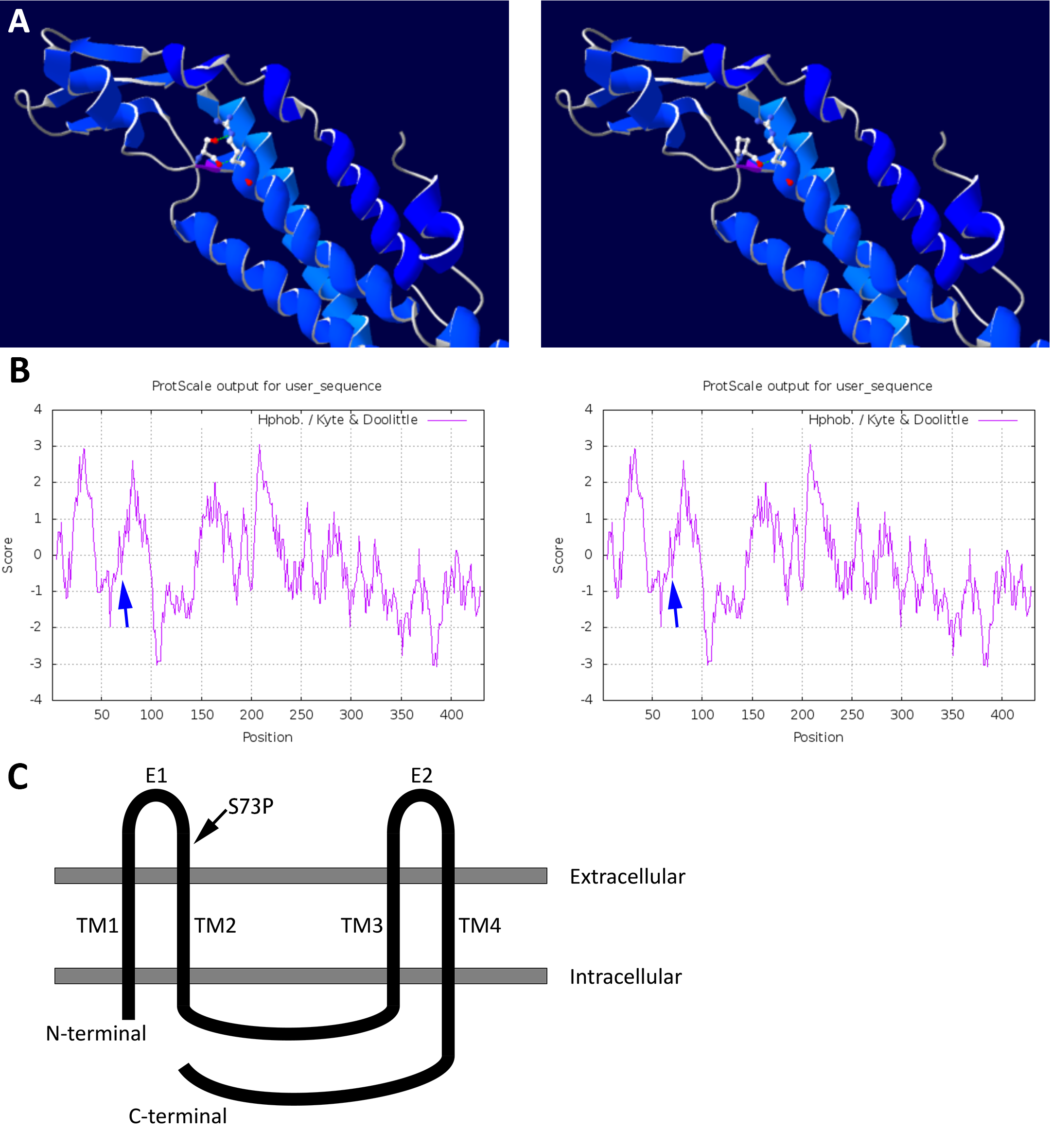
**

**Figure S2.** 3D-model, hydrophobicity, and transmembrane structure of wildtype and mutant Cx50 proteins. A, 3D-model structure of wildtype and mutant Cx50 proteins predicted by SWISS-MODEL. B, Hydrophobicity of wildtype and mutant Cx50 protein predicted by Protscale; the blue arrow indicates the score difference at position 73. C, Transmembrane structure of the Cx50 protein; the black arrow represents the mutation site.

**
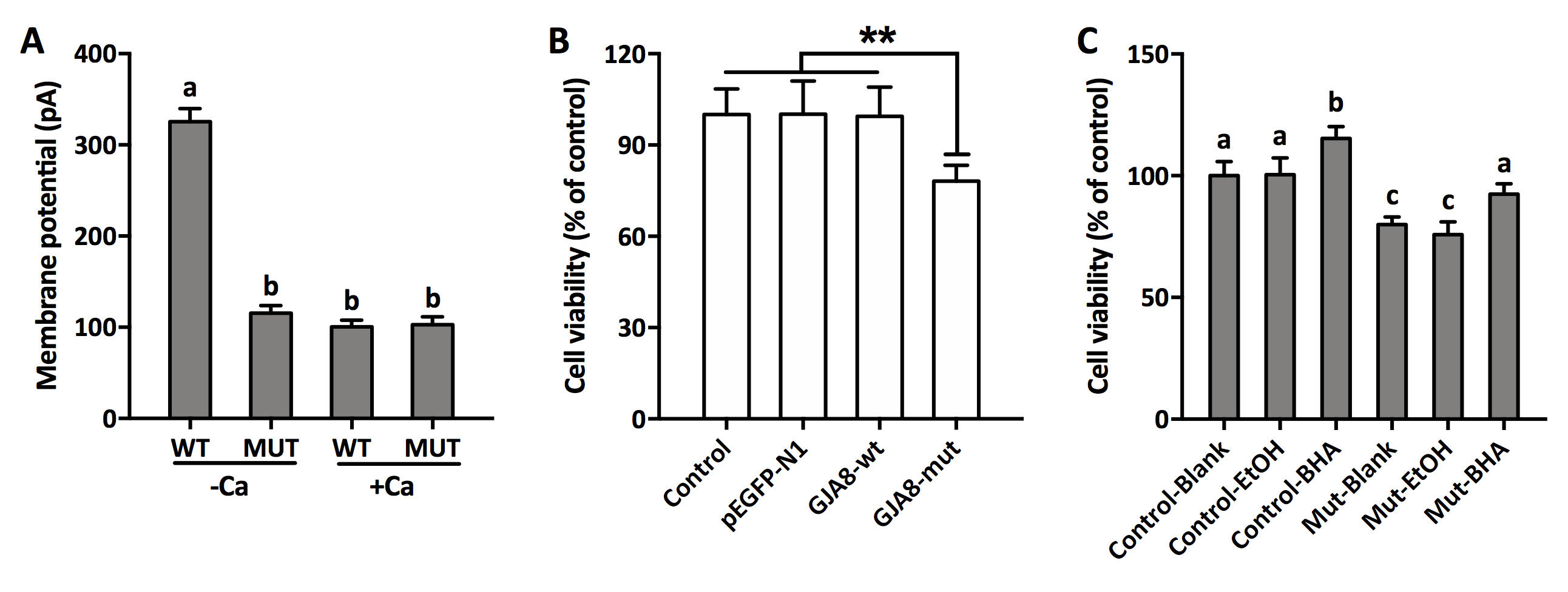
**

**Figure S3.** Membrane potential and cell viability of transfected cells. A, Membrane potential of wildtype and mutant Cx50-transfected cells with or without Ca^2+^. B, Cell viability of control cells, pEGFP-N1-transfected, GJA8-wt-transfected and GJA8-mut-transfected cells by the CCK-8 assay. C, Cell viability of transfected cells treated with BHA or vehicle. A significant difference is indicated at the *p* < 0.01 (**) level. Different lowercase letters on the bars indicate significant differences at the *p* < 0.05 level. Each value represents the mean ± SD (*n* = 3).

**
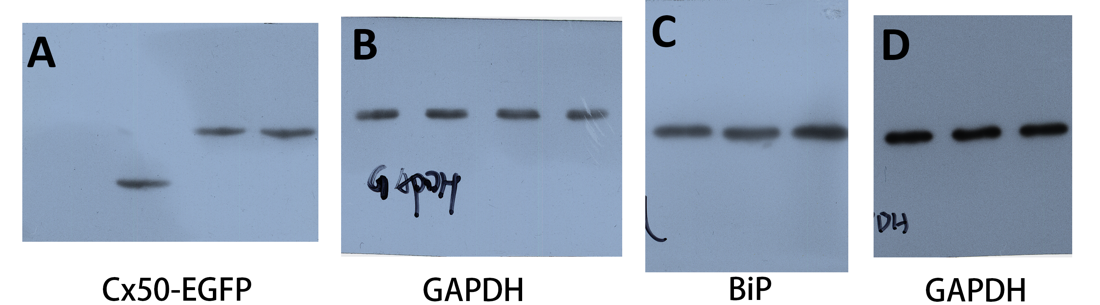
**

**Figure S4.** Original Western blot digital images for Figure 2A and Figure 6A. A, Original image for Figure 2A of the protein using the primary anti-EGFP antibody. Lane 1, control; lane 2, pEGFP-N1-transfected; lane 3, GJA8-wt-transfected; lane 4, GJA8-mut-transfected. B, Original image for Figure 2A of the protein using the primary anti-GAPDH antibody. Lane 1, control; lane 2, pEGFP-N1-transfected; lane 3, GJA8-wt-transfected; lane 4, GJA8-mut-transfected. C, Original image for Figure 6A of the protein using the primary anti-BiP/Grp78 antibody. Lane 1, control; lane 2, GJA8-wt-transfected; lane 3, GJA8-mut-transfected. D, Original image for Figure 6A of the protein using the primary anti-GAPDH antibody. Lane 1, control; lane 2, GJA8-wt-transfected; lane 3, GJA8-mut-transfected. Detailed molecular weights of each western blot image were shown in Figure 2A and Figure 6A.
